# Damage control surgery by keeping the abdomen open during pregnancy: favorable outcome, a case report

**DOI:** 10.1186/1749-7922-4-33

**Published:** 2009-09-24

**Authors:** Wojciech Staszewicz, Michel Christodoulou, François Marty, Vincent Bettschart

**Affiliations:** 1 Department of Surgery, Mid-Valais Hospital Center, Sion, Switzerland; 2 Department of Gynecology, Mid-Valais Hospital Center, Sion, Switzerland

## Abstract

**Background:**

Acute abdomen in advanced pregnancy is one of the most challenging surgical situations. In life-threatening situations, despite optimal management, foetus distress and preterm delivery may occur. Although laparostomy is a useful treatment of abdominal sepsis, its successful management has not been reported previously in pregnant women.

**Case:**

30-year-old woman at 23 week of pregnancy was investigated for non-specific abdominal pain. Surgical exploration revealed extensive ischemic bowel necrosis. Multiple segmental resections were performed and abdomen was left open with vacuum assisted dressing, maintained for 48 hours. At the third surgical look the continuity was restored and abdominal wall closed. The foetal condition stayed unperturbed under pharmacologic tocolysis. Pregnancy was carried to full term delivery.

**Conclusion:**

Open abdomen strategy can be successfully applied in pregnant woman.

## Background

Acute abdominal pain in advanced pregnancy remains a diagnostic and management challenge. During pregnancy the usual clinical presentation is masked by gravid uterus and physiological changes. Imaging procedures can rarely help to resolve a diagnostic dilemma because of modified abdominal anatomy and limits in x-ray techniques use [1]. For these reasons the rate of accurate preoperative diagnosis is still considerably lower than in non-pregnant patients. In many cases early laparoscopy is the best both diagnostic and therapeutic tool [2].

For the most frequent acute abdomen causes including acute appendicitis, cholecystitis, mechanical obstruction and gastric ulcer perforation standard surgical management gives relatively good outcomes with overall 6% of miscarriage, 2.5% of foetus lost and less then 4% of premature labour rate [3]. The maternal mortality rate is comparable to non-pregnant surgical patients. Long-term follow-up of laparoscopic surgery proves the safety and efficiency of this technique in pregnant woman [4].

However, the decision to operate is often delayed during pregnancy. This is probably the first reason of high foetal or mother morbidity. The recommendation in these situations is to manage the surgical problem regardless of the pregnancy using the same surgical strategy as for non-pregnant patient [5].

## Case presentation

A 30-year-old woman was admitted to the emergency department at 23 week of her second pregnancy for non-specific abdominal pain. She was known for previous minor abdominal surgery including mesenteric cyst excision and vesicoureteral reflux surgery in childhood followed by laparoscopic adhesiolysis 10 years later. She had no fever and no vomiting or constipation history. Biological tests including RBC, WBC, C-reactive protein, bilirubin, pancreatic enzymes and serum lactates were also still normal during 48 hours of observation. The initial imaging investigations by abdominal and pelvic ultrasound showed no intra-abdominal abnormalities and the plain abdominal x-ray at 48 hours revealed only some very slightly dilated small bowel loops. The foetus status in ultrasound was normal.

**Figure 1:**
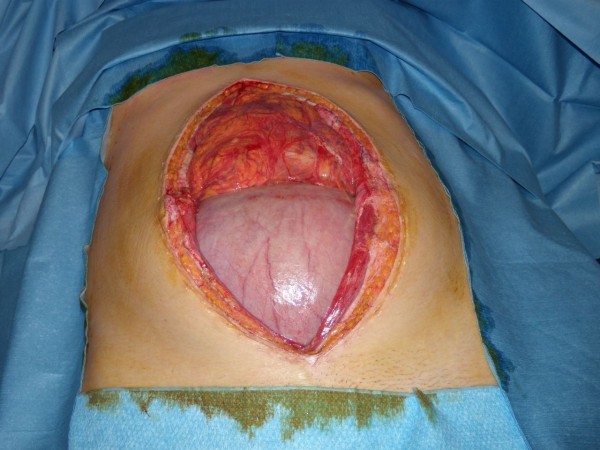
**Open abdomen**. The gravid uterus is seen in the inferior half of the laparostomy.

**Figure 2:**
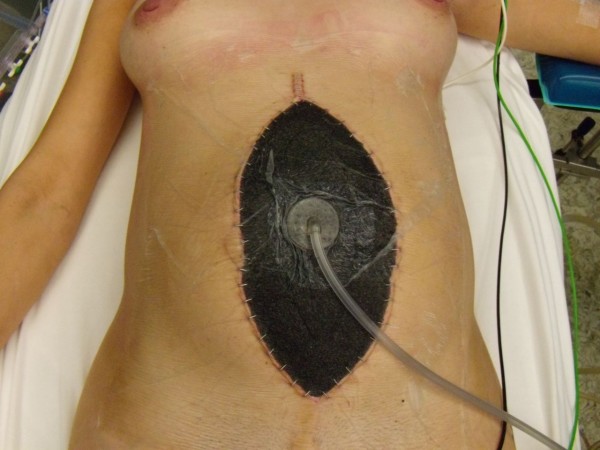
**Open abdomen with vaccum dressing**.

**Figure 3:**
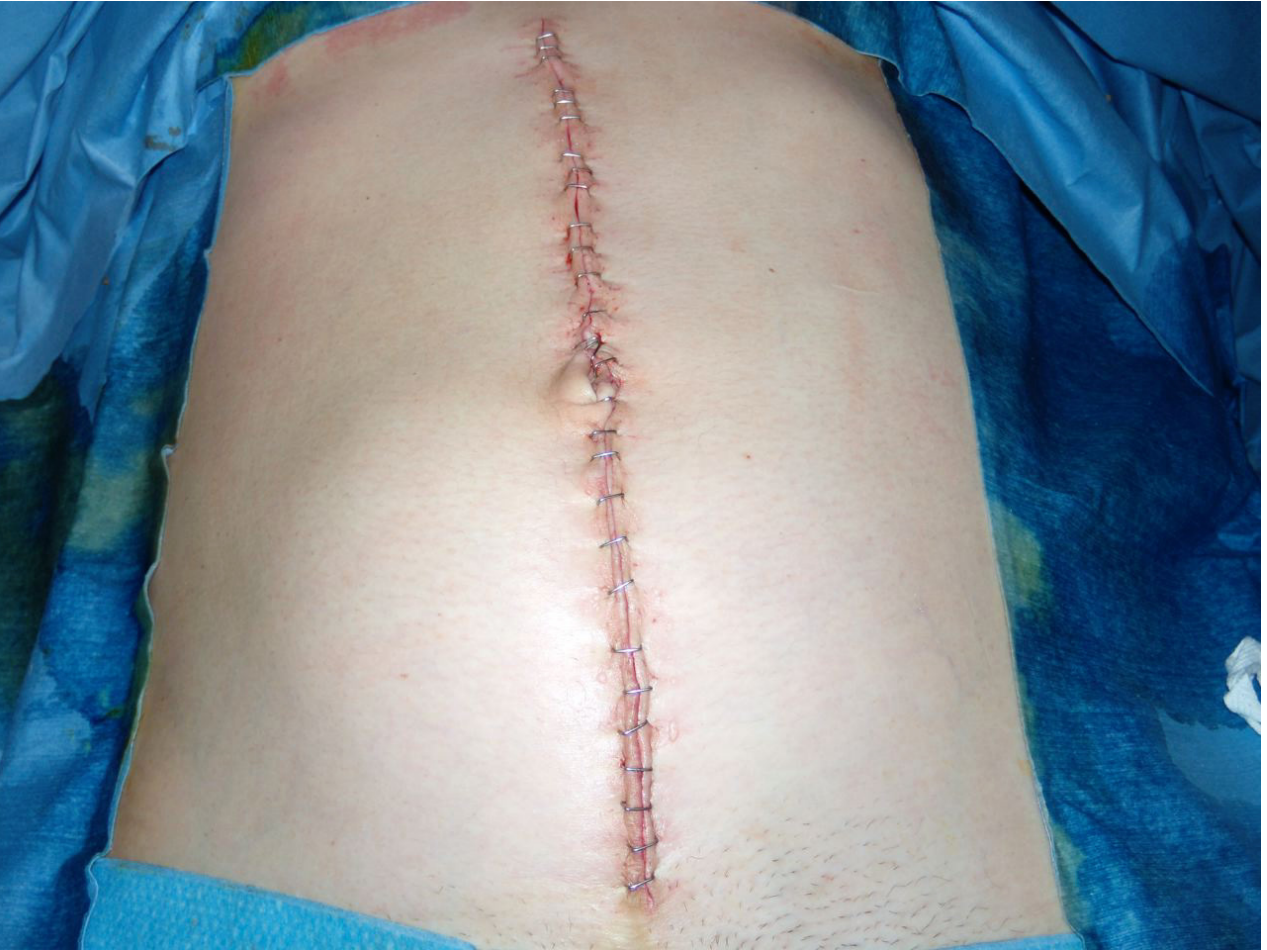
**Abdomen primarily closed after 48 hours of laparostomy**.

Persistence of pain not relieved with strong analgesics conducted to laparoscopic exploration despite the absence of biological or radiological abnormality. Laparoscopy revealed massive necrotic lesions of the small bowel with rare viable segments in discontinuity. After conversion to laparotomy multiple segmental resections were performed, potentially viable bowel segments were closed by stapling and abdomen was left open with vacuum assisted dressing in the aim to asses the viability of remaining bowel after 24 and 48 hours (figures 1, 2). The vacuum abdominal closure was done using a negative pressure therapy system ([NPWT] V.A.C.^® ^Therapy™, KCI Inc.) with 125 mmHg continuous negative pressure. At the second and third surgical look some intestinal segments required subsequent additional resections. Eventually, after 48 hours of open abdomen management, the intestinal continuity was restored leaving 110 cm of viable small bowel. Abdominal wall was primary closed without aponeurotic defect (figure 3).

During the two days where the abdomen was left open, optimal foetal and mother conditions were maintained by intensive care procedures including sedation, mechanical ventilation, liquid resuscitation, adapted parenteral nutrition and pharmacologic tocolysis by hexoprenaline.

The patient left the intensive care unit on 9^th ^postoperative day. Complete recovery requires in-hospital and ambulatory nutritional support for short bowel syndrome. Pregnancy was uneventfully carried to full term vaginal delivery.

## Conclusion

Open abdomen management has become a commonly adopted strategy in severe surgical conditions. Critical intra-abdominal infection, blunt or open trauma, intestinal ischemia and abdominal hypertension are typical indications to leave the abdomen open. It is also the treatment of abdominal compartment syndrome. The rare cases of laparostomy use in pregnant women previously reported haven't been successful in terms of foetus or mother issue [6]. The authors found only one case report of favorable outcome after laparostomy as a treatment of wound dehiscence in pregnant women [7].

In the present case leaving the abdomen open was a deliberate intraoperative decision. We adopted the principles of damage control surgery consisting of planned subsequent delayed explorations after the primary debridement and necrotic bowel resections.

It was shown that temporary dressing with vacuum pack is a safe, well tolerated technique [8]. The disadvantage of laparostomy is the difficulty of the subsequent fascial closure. Abdominal sepsis and trauma seems associated with higher rate of fascial closure failure and consecutive incisional hernia. Among many techniques developed for open abdomen management, vacuum assisted closure (VAC) allows currently the best results in term of primary abdominal wall closure [9]. In some series, using VAC protocols, complete fascial closure rate was achieved in 100% [10]. In abdomen with constantly growing gravid uterus and low intra-abdominal pressures requirements, primary closure appears to be a particularly challenging task. It is nevertheless a key endpoint in a pregnant woman, in order to protect the foetus and to assure a vaginal delivery.

The present case report contributes to the rational that decision making in severe abdominal surgical emergency in pregnant women should respect the same principles and use the same techniques as in non-pregnant patient. The decision process should not be delayed by pregnancy. The management of acute abdomen by laparostomy during pregnancy is feasible, and may be associated with a good outcome for both the mother and the child.

## Consent

Written informed consent was obtained from the patient for publication of this case report and any accompanying images.

## Conflict of interests

The authors declare that they have no competing interests.

## Authors' contributions

WS and MC contributed equally to this work; WS and MC drafted the paper; WS wrote, FM critically revised and VB critically revised the paper with an important conceptual and editorial input. All authors read and approved the final manuscript.
